# Association between antibiotic use and the risk of rheumatoid arthritis: A protocol for a systematic review

**DOI:** 10.1097/MD.0000000000019155

**Published:** 2020-02-28

**Authors:** Tingting Meng, Shibin Zhang, Dawei Wang, Haijun Zhang, Zhongyang Song, Shengdong Li, Min Chen, Chunzhi Tang

**Affiliations:** a Faculty of Chinese Medicine, Macau University of Science and Technology, Macau University of Science and Technology, Avenida Wai Long, Taipa, Macau; b Shunde Hospital of Guangzhou University of Chinese Medicine, Shunde District Hospital of Chinese Medicine of Foshan City, Foshan, Guangdong; c Department of Orthopaedics, Gansu Second Provincial People's Hospital; d Clinical Medical College of Traditional Chinese Medicine, Gansu University of Chinese medicine, Lanzhou, Gansu; e Department of Rheumatology, The Second Hospital of Yinzhou, Ninbo Zhejiang, China; f The State Key Laboratory of Quality Research in Chinese Medicine, Macau University of Science and Technology, Avenida Wai Long, Taipa, Macau; g Clinical Medical College of Acupuncture, Moxibustion and Rehabilitation, Guangzhou University of Chinese Medicine, Guangzhou, Guangdong, China.

**Keywords:** antibiotic, meta-analysis, protocol, rheumatoid arthritis, systematic review

## Abstract

**Background:**

The potential association between antibiotic use and the risk of rheumatoid arthritis (RA) has drawn significant attention from clinicians and researchers in recent years due to the wild usage of antibiotic. This study aimed to perform a systematic review and meta-analysis of the literature to determine if antibiotic use is associated with an increased risk of RA, so as to provide an important reference for clinical decision-making.

**Methods:**

Case-control and nest case-control studies of assessing whether antibiotic use is associated with the onset of RA will be identified in searches of 4 databases from their inception to August 2019. All data were assessed and extracted by 2 authors independently. The Newcastle–Ottawa scale was used to assess the quality of the selected studies. Manager Software 5.3 from Cochrane Collaboration (London, UK) and Stata 15.1 (Stata Corp, College Station, TX) will be used to conduct meta-analysis, determining pooled odds ratios and evaluating heterogeneity between studies.

**Result:**

The results of this systemic review and meta-analysis will be submitted to a recognized journal for publication.

**Conclusion:**

This systemic review and meta-analysis will determine if antibiotic use is associated with an increased risk of RA. We hope this study can make a definitive conclusion for the association.

## 1 Introduction

Antibiotics are wildly used to treat a wide range of bacterial infections for example, respiratory, gastrointestinal, and urinary tract infections.^[1]^ Around 30% of all patients registered in primary care receive at least 1 antibiotic prescription per year in the UK.^[2]^ Antibiotics targeting bacterial pathogens, ln the same time they disturb the gut microbiota. Which play important role in modulating host metabolism and immunity.^[3–5]^ Importantly, the microbiota can be influenced by numerous factors, with antibiotic treatment suggested as among the most significant.^[6]^

Antibiotic treatment can reduce the gut, oral, and skin microbiota, leading to an immediate reduction in microbial abundance and species diversity. A number of studies indicated that antibiotic usage may appears to increase the risk of autoimmune conditions, including type 1 diabetes, autoimmune live disease, inflammatory bowel diseases, and juvenile idiopathic arthritis.^[7–9]^

Rheumatoid arthritis (RA) is a chronic autoimmune inflammatory disease, its etiology is not clear. Genetics, smoking, and hormones have all been implicated.^[10–12]^ However, the emerging idea of a microbial trigger (infections of a specific body part by a specific pathogen) has been investigated in greater depth recently. In the UK, 1 study indicated that there were positive associations between bacteria such as *Porphyromonas gingivalis* and Aggregatibacter actinomycetemcomitans infection and incident RA.^[13,14]^ Several animal experiments have showed the relationship between microbiota and the development of inflammatory arthritis. Germ-free mouse models do not develop inflammatory arthritis, and certain bacteria (eg, segmented filamentous bacteria, a mouse-specific taxa) have been shown to induce inflammatory TH17 responses, which may drive associated immune-mediated pathology and joint destruction.^[15,16]^ Antibiotics have been shown to significantly perturb the gut and urinary microbiome, with trials showing substantial microbial shifts in the gut lasting up to a year after treatment periods of at least 1 week.^[17]^ Even though recent studies had known that the usage of antibiotics could perturb the microbiome. While the association has not been adequately investigated.

At present, meta-analysis has been increasingly regarded as a high-quality method for determining research evidence to support treatment and clinical decisions.^[18,19]^ This study aimed to perform a systematic review and meta-analysis of the literature to determine if antibiotic use is associated with an increased risk of RA.

## 2 Methods

### 2.1 Study registration and ethics

This protocol has been registered at PROSPERO (registration number: CRD42019147749; http://www.crd.york.ac.uk/PROSPERO). Data from individual patients will not be used in this systemic review and no privacy will be involved. So the ethical approval is not necessary.

### 2.2 Selection criteria

#### 2.2.1 Type of study

We will include case-control studies including nest case-control studies; the language of the literature will not be limited. Cohort studies, cross-sectional studies, case reports, review articles, conference abstracts, editorials, letters, and expert opinions will be excluded.

#### 2.2.2 Participants

People were diagnosed as RA complied with Read Codes, Characteristics such as age, sex, and ethnicity were not restricted.

#### 2.2.3 Exposure

The case group must be Oral antibiotics at least 1 year, topical antibiotics is not considered as exposure, frequency of daily medication, types of antibiotics will not be restricted. The control group is participants did not use antibiotics or used antibiotics for a period of time (less than 1 year) or topically.

#### 2.2.4 Outcomes

The outcomes are odds ratio (OR) of the incidence rate of RA.

### 2.3 Search strategy

We will search 4 databases including PubMed, Web of Science, EMBASE, and Cochrane Database of Systematic Reviews, from their inception to August 20th, 2019. The search term will be “Anti-bacterial agents,” “Antibiotics” and “rheumatoid arthritis,” “rheumatism,” “atrophic arthritis.” MeSH terms “Antibiotics,” ”Antibacterial agents,” “rheumatoid arthritis” or “rheumatism.” We will also conducted a search of reference lists from relevant review articles and the final included studies for additional sources. Details of the strategies used to search databases are shown in the Supplementary Materials.

### 2.4 Data extraction

Data were extracted by 2 authors independently. All the differences were settled by discussion between the 2 researchers. If we cannot reach an agreement, we will consult a third reviewer. Data extraction included the basic information of the trial (name of the first author, publish year), research basic Information (Patient information, types of antibiotics, period of antibiotic use, dosage of antibiotics), outcomes (OR of RA incidence), relevant important variables.

### 2.5 Quality assessment

Assessment of the risk of bias of the included studies was performed independently by 2 authors and was ranked as high, low, or uncertain. Possible discrepancies were adjudicated by the other study-team members. All included studies were case–control studies. Bias risk assessment was conducted using the Newcastle–Ottawa scale,^[20]^ which assesses the selection of study groups, comparability of study groups, and exposure measurement. Using that scale, 9 = maximum score, >6 is relatively high quality, 5 to 6 is fair quality, <5 is poor quality.

### 2.6 Statistical analysis

#### 2.6.1 Meta-analysis

Meta-analysis was performed using Review Manager Software 5.3 from Cochrane Collaboration (London, UK) and Stata 15.1 (Stata Corp, College Station, TX). The outcome measures of interest for the meta-analysis will be OR of RA incidence. Pooled OR with 95% confidence intervals were calculated for dichotomous variables (disease incidence).

#### 2.6.2 Measures for heterogeneity

The degree of heterogeneity (*I*^2^) of each outcome will be analyzed using the Chi-squared test, with no significance designate by *P* > .05. *I*^2^ < 50% indicates low heterogeneity of the data and the fixed model adopted for a meta-analysis; otherwise the random effects model will be used. If substantial heterogeneity is detected, subgroup or sensitivity analysis and meta-regression is applied to explore the causes of heterogeneity. If the sources of heterogeneity could not be determined, we adopt descriptive analysis.^[21]^

#### 2.6.3 Publication bias

Funnel plot and Egger test will be applied to evaluate the existence of publication bias.^[22]^

## 3 Discussion

A number of case-control studies have shown the Association between antibiotic use and the risk of RA. While the participants included in the studies were relatively small and no article has found to summarize the existing evidence. Due to the simple was small, we cannot judge whether there is association antibiotic use and the risk of RA. Therefore, this systemic review and meta-analysis we conduct so as to provide reliable evidence. If they really have the association, in the future may we should carefully use antibiotic for a long time, especially for patients with RA.

This protocol has been registered, we will strictly execute according to the Cochrane Handbook for Systematic Reviews of interventions and is presented per the preferred reporting items for systematic reviews and meta-analyses guideline.

However, several limitations may occur in this review. First, for the reasons of competence we only searched 4 international database that will be lead to selection bias. Second, for most primary studies, the type of antibiotic was different and some studies may combine 2 or more types of antibiotic, this may cause heterogeneity. The treatment frequency, duration of treatment, and individual difference for example renal function and liver function are varied, which may also cause heterogeneity. Third, this research is based on researches presently; the emergence of new researches in the future may have an impact on current results, so we will update the study in the future. In conclusion, we hope this study can exactly find the association between antibiotic use and the risk of RA.

## Author contributions

**Conceptualization:** Tingting Meng and Shibin Zhang.

**Data curation:** Tingting Meng.

**Funding acquisition:** Min Chen.

**Methodology:** Dawei Wang.

**Project administration:** Min Chen.

**Protocol draft:** Tingting Meng and Shibin Zhang.

**Software:** Shengdong Li, Zongyang Song and Haijun Zhang.

**Study design:** Tingting Meng, Shibin Zhang and Chunzhi Tang.

**Validation:** Min Chen and Chunzhi Tang.

**Writing – original draft:** Tingting Meng.

**Writing – review & editing:** Shibin Zhang.
